# Metallic foreign object in postoperative chest radiograph?

**DOI:** 10.4103/0019-5049.76576

**Published:** 2011

**Authors:** Dheeraj Arora, Abhishek Bansal, Yatin Mehta

**Affiliations:** Medanta Institute of Critical Care and Anaesthesiology, Medanta — The Medicity, Gurgaon, India

**Figure 1:**
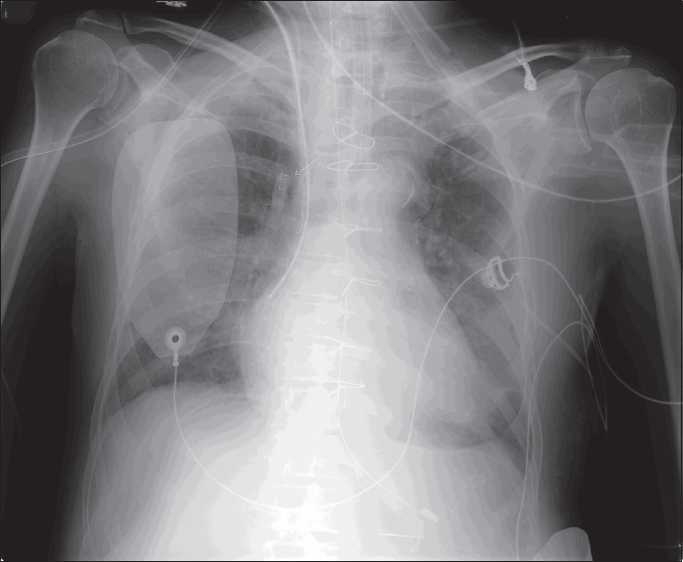
Chest radiograph showing metallic object lateral to the right sternal border

Sir,

A 63-year-old male underwent beating heart coronary artery bypass surgery at our institute. Post procedure, the patient was shifted to Cardiac Recovery with endotracheal tube, Swan Ganz catheter and femoral arterial cannula in situ. The procedure was completed uneventfully, with complete counts of all the materials used by the surgeon for the surgery. The on-duty resident found a radio opaque coiled wire like shadow lateral, to the right sternal border in the third intercostal space (ICS) on a post operative chest radiograph anteroposterior view [Figure 1]. The presence of this unique foreign body raised a suspicion.

**Figure 2:**
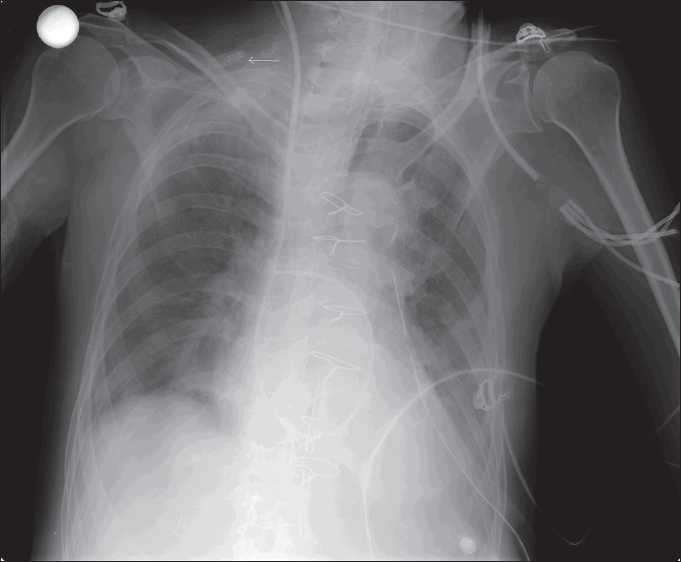
Chest radiograph showing metallic object above the right clavicle

At the first instance the possibility of a metallic object being left in was thought of, but it was ruled out on the assurance of the surgeon and the assistant operating staff. On physical examination no metallic object was found on the patient’s chest. The digital chest X-ray was repeated. The repeat radiograph showed the same foreign body lying on the right side of the neck, above the clavicle, with a clear demarcation of the pilot balloon [Figure 2]. Thus, diagnosis of pilot balloon of the tracheal tube and its spring loaded valve falsely appearing as a foreign body was made.

Artefacts can commonly be seen in a chest X-ray film. However, the familiarity and knowledge of various surgical materials like vascular clips, stents, sternal wires, needles, intra-aortic balloon catheter tips and so on, can help clinicians in differentiating these from the artefacts. Also the knowledge of some common radio opaque structures, which may appear as artefacts should also be kept in mind. Chakarabarthy and others reported radio opaque rings seen in the post off-pump coronary artery bypass chest radiograph as epidural connectors.[1] Arora and others reported the hearing aid of a patient appearing as a pacemaker on an X-ray.[2] As any surgical mishap such as wires, shunts, sponges and so on can have catastrophic consequences and medicolegal implications,[3] early recognition of such events is warranted, using other imaging modalities if need be. Adequate knowledge and cautious reading and reporting of X-rays may prevent unnecessary interventions. The pilot balloon may be secured along with the tracheal tube, particularly in patients undergoing cardiac surgery or requiring prolonged ventilation, so that it does not mislead the clinician.
